# Supplementary material for: Synthesis and Cytostatic Effect of 3’-deoxy-3’-C-Sulfanylmethyl Nucleoside Derivatives with d-xylo Configuration
Source: Molecules. 2019 Jun 10;24(11):2173. doi: 10.3390/molecules24112173 (PMC6600393; doi:10.3390/molecules24112173)
Supplement: Supplementary file 1 [file molecules-24-02173-s001.pdf]

Supporting Information

Synthesis and cytostatic effect of 3'-deoxy-3'-*C*-sulfanylmethyl nucleoside derivatives with D-*xylo* configuration

Miklós Bege[1], Alexandra Kiss[2], Máté Kicsák[1], Ilona Bereczki[1], Baksa Viktória[2], Gábor Király[2], Gábor Szemán-Nagy[2], M. Zsuzsa Szigeti[2], Pál Herczegh[1], Anikó Borbás[1],*

1: Department of Pharmaceutical Chemistry, University of Debrecen, 4032 Debrecen, Egyetem tér 1, Hungary

2: Department of Biotechnology and Microbiology, University of Debrecen, Hungary

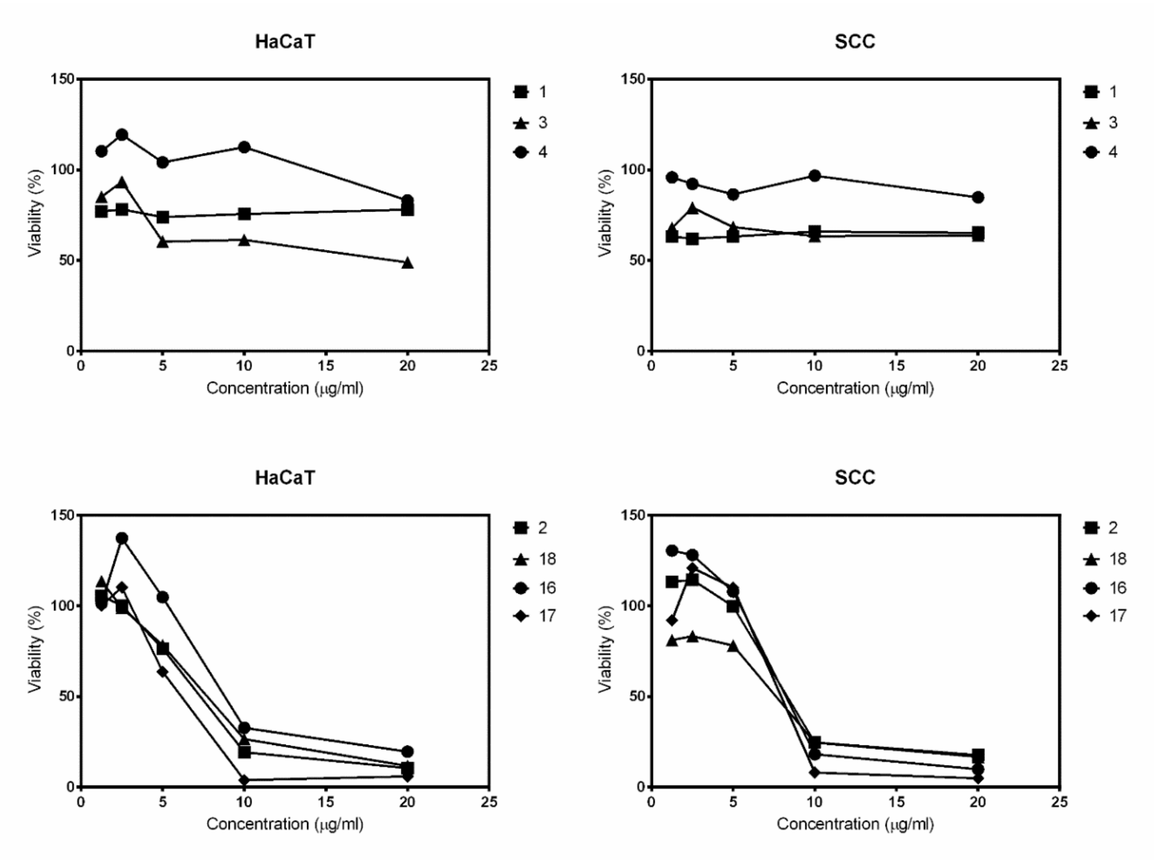

Figure S1: Concentration dependent effect of compounds **1**, **3** and **4** (upper diagrams) and compounds **2**, **16**, **17** and **18**. on cell viability.

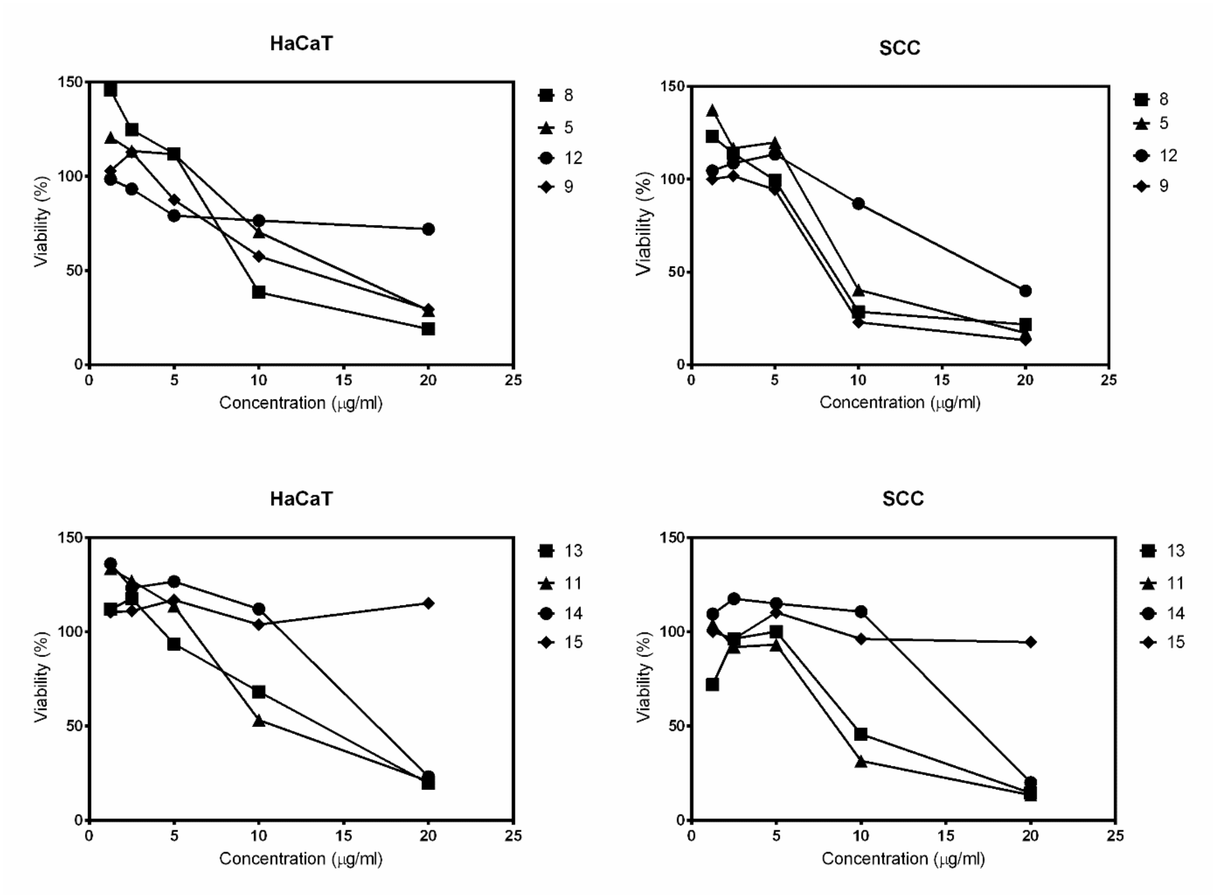

Figure S2: Concentration dependent effect of compounds **5**, **8, 9,** and **12** (upper diagrams) and compounds **11, 13, 14** and **15** on cell viability

^{1}H and ^{13}C NMR spectra of compounds

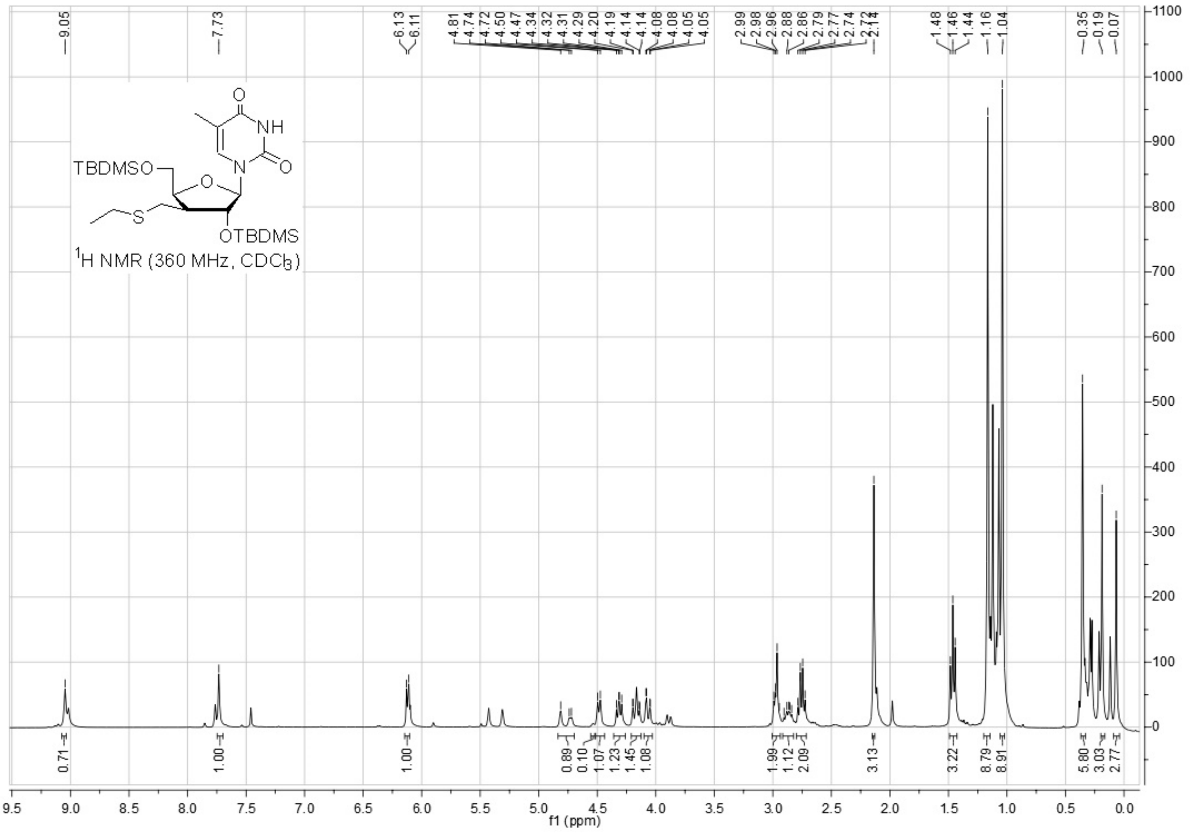

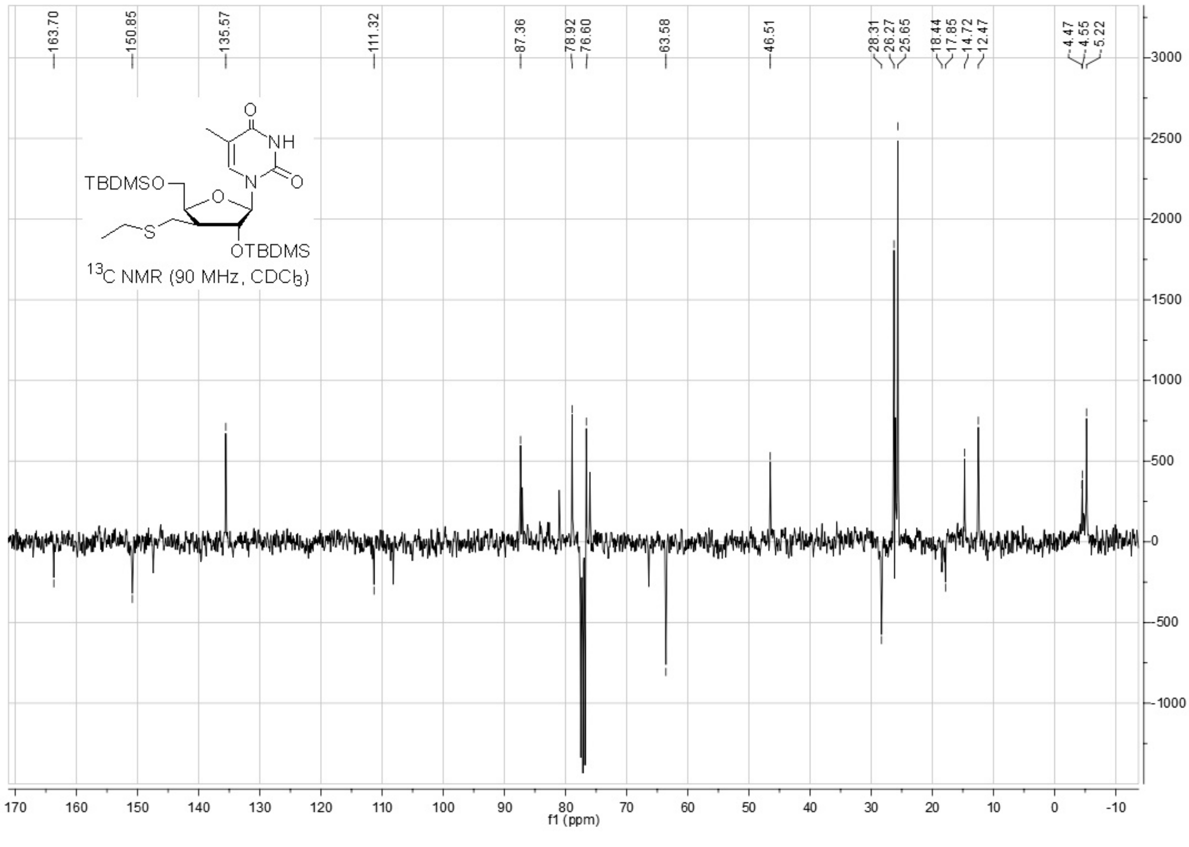

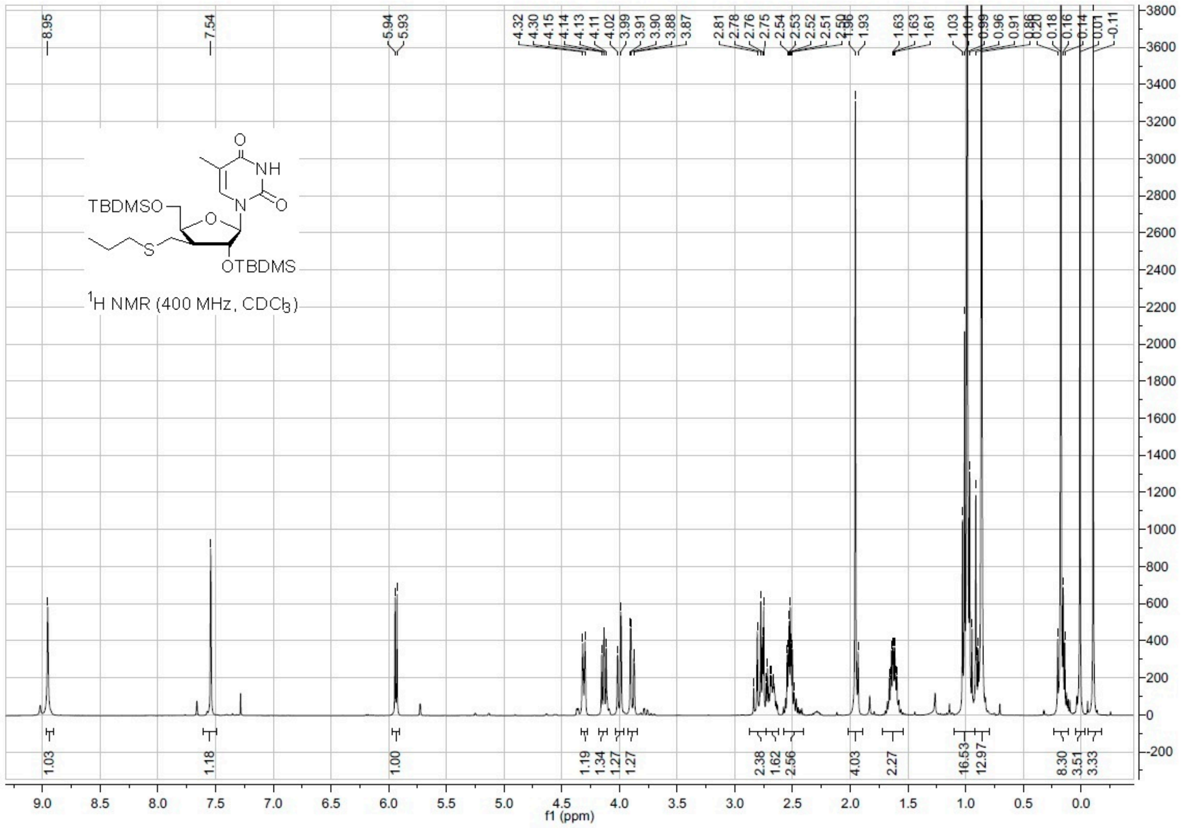
TBDMSO
S
OTBDMS
O
NH
N
O
O
1H NMR (400 MHz, CDCl3)
f1 (ppm)

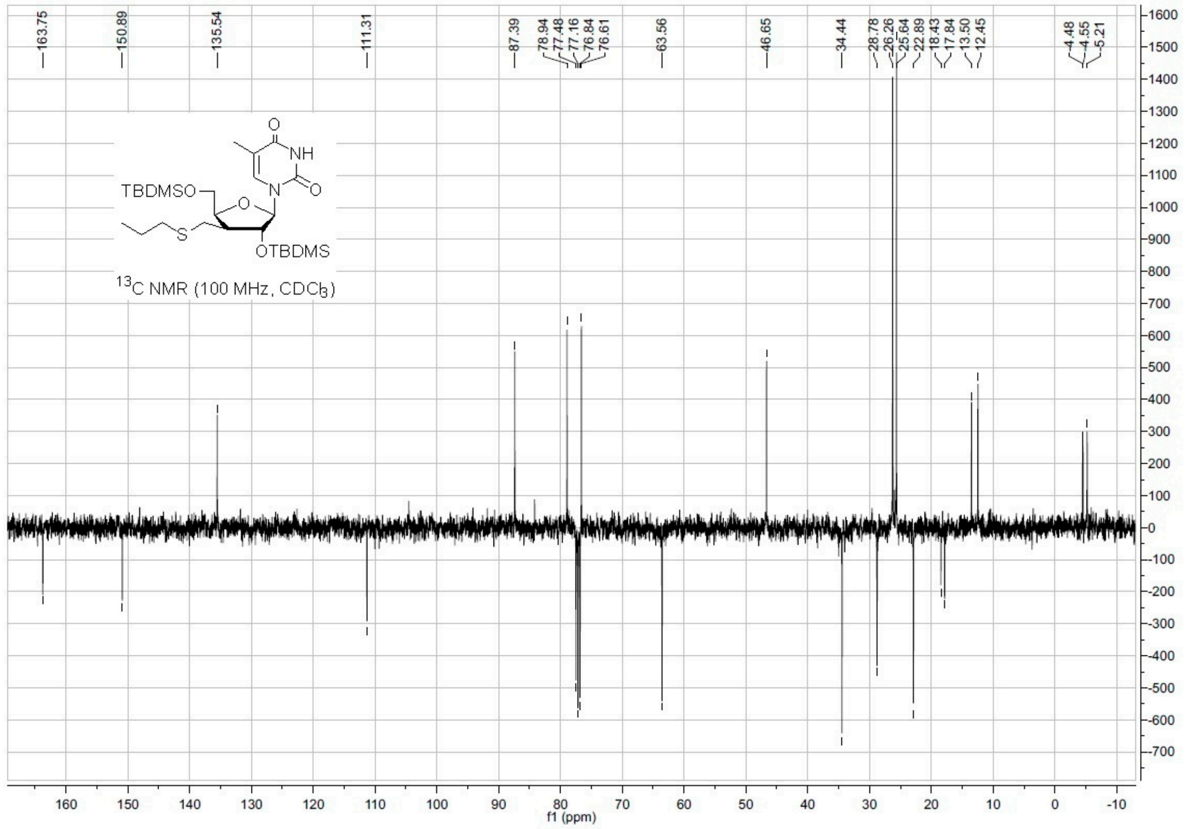
TBDMSO
S
OTBDMS
O
NH
N
O
O
13C NMR (100 MHz, CDCl3)
f1 (ppm)

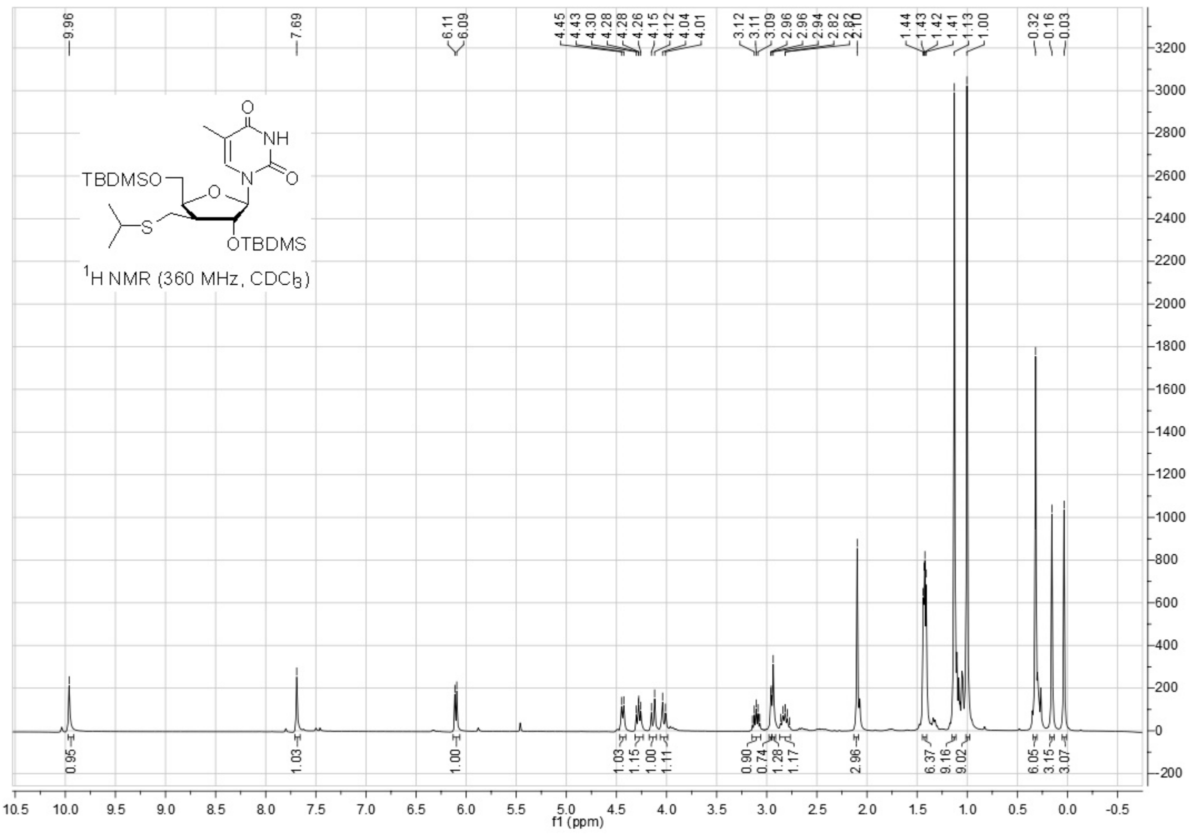

TBDMSO
NH
O
S
OTBDMS
1H NMR (360 MHz, CDCl3)
f1 (ppm)

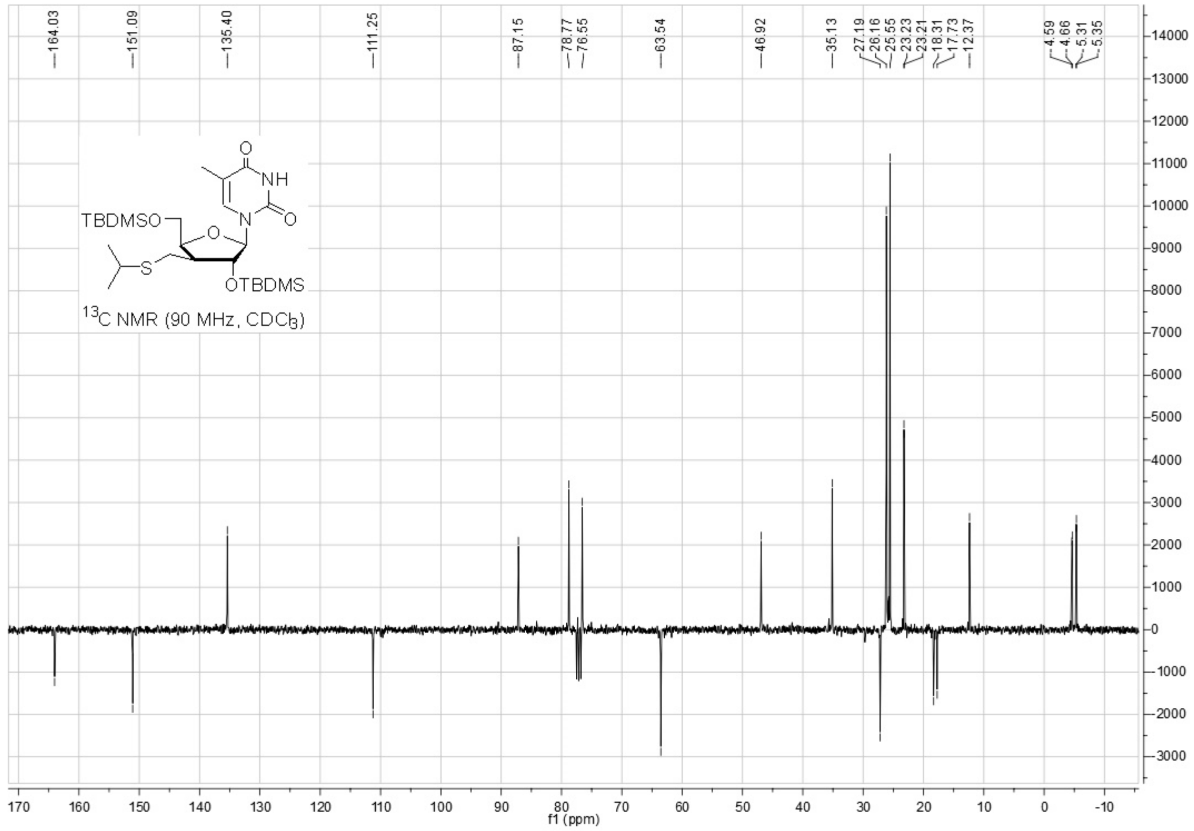

TBDMSO
O
NH
N
O
S
OTBDMS
^{13}C NMR (90 MHz, CDCl3)
f1 (ppm)

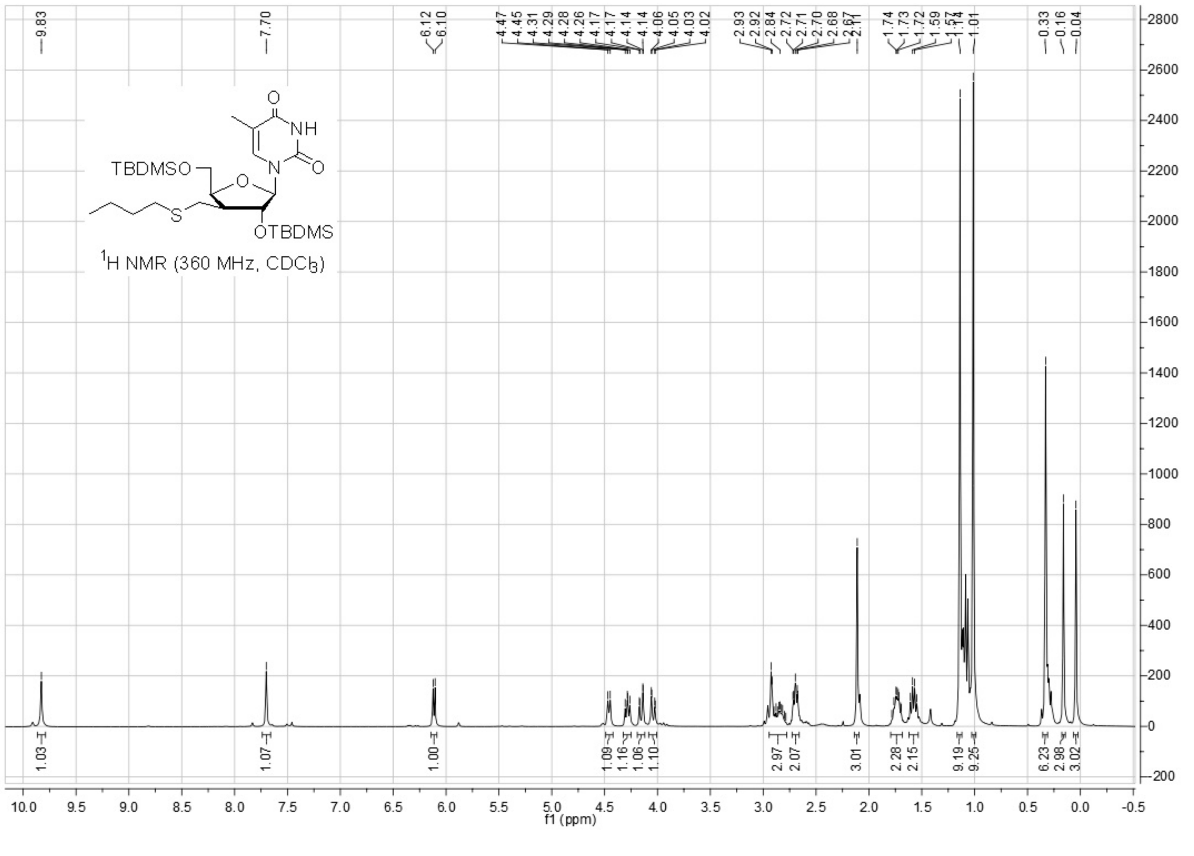

TBDMSO
O
NH
N
O
S
OTBDMS
^{1}H NMR (360 MHz, CDCl3)
f1 (ppm)

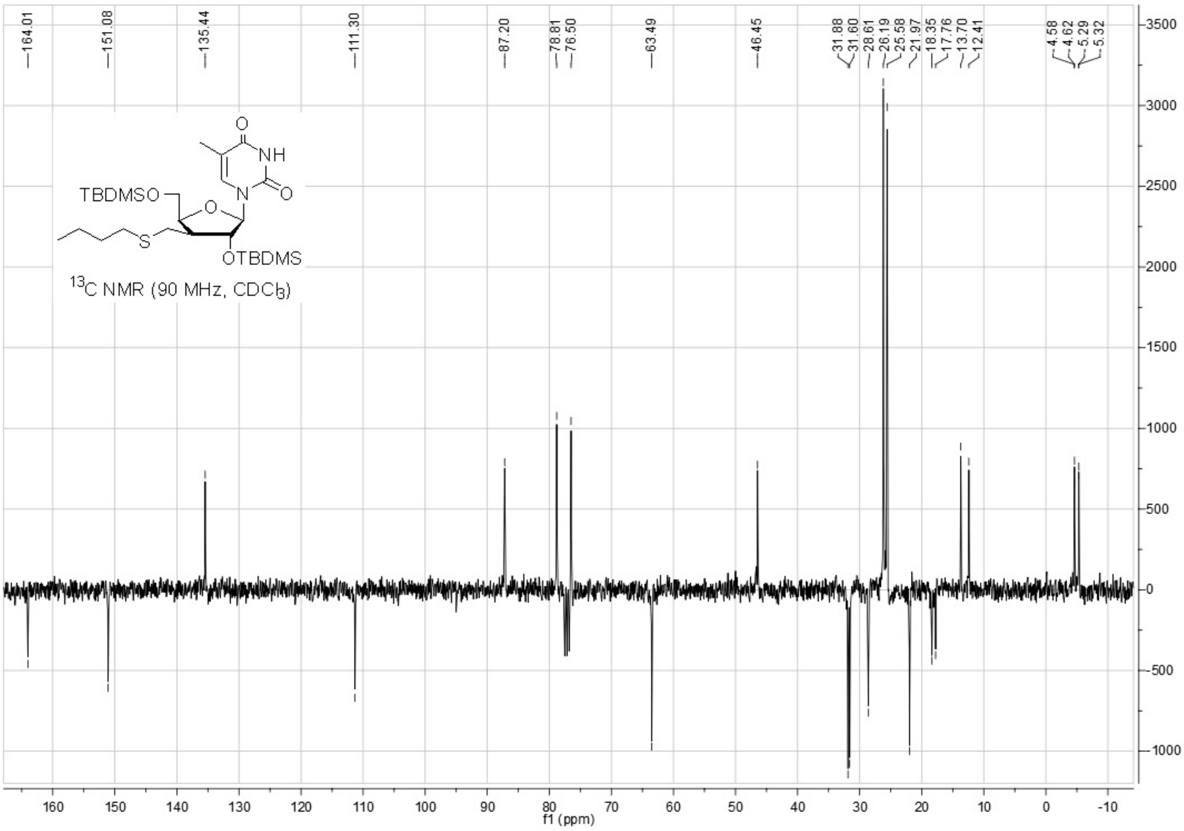

164.01
151.08
135.44
111.30
87.20
78.81
76.50
63.49
46.45
31.88
31.60
28.61
26.19
25.58
21.97
18.35
17.76
13.70
12.41
-4.58
-4.62
-5.29
-5.32
TBDMSO
NH
O
N
S
OTBDMS
^{13}C NMR (90 MHz, CDCl$_3$)
f1 (ppm)

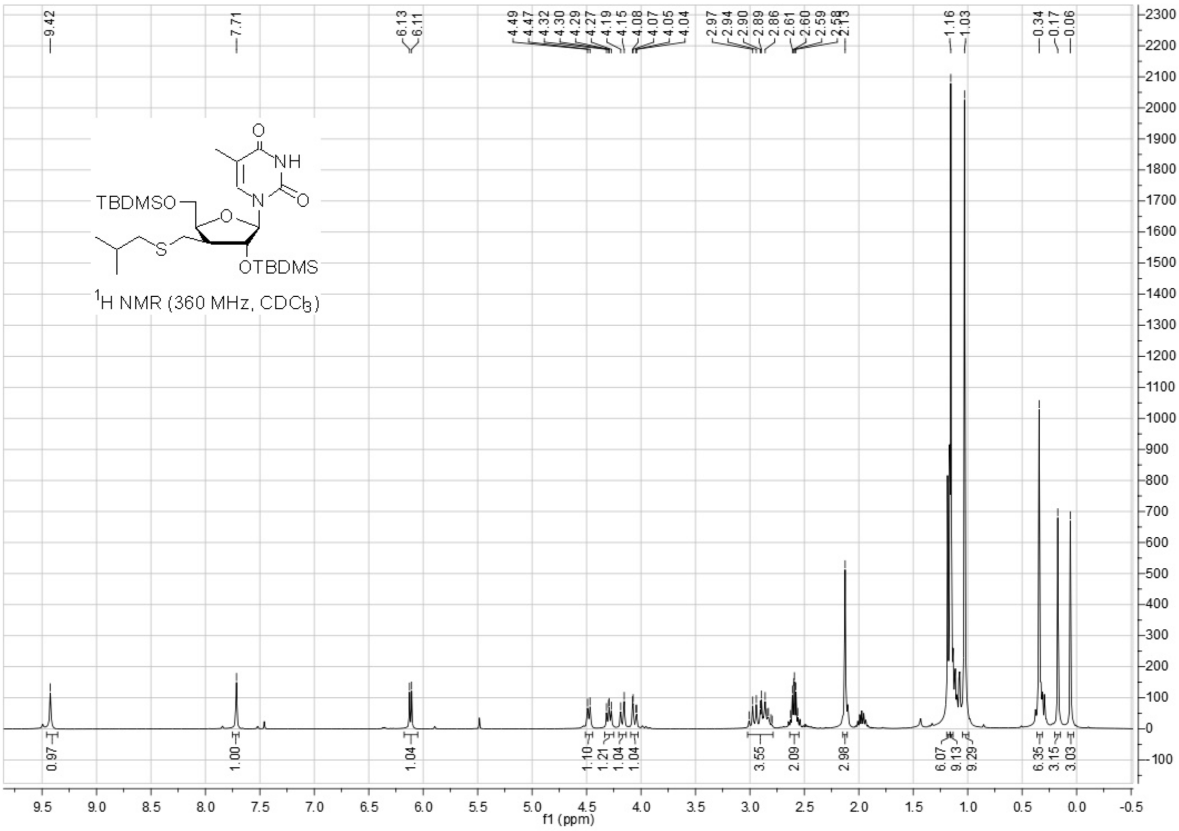

9.42
7.71
6.13
6.11
4.49
4.47
4.32
4.30
4.29
4.27
4.19
4.15
4.08
4.07
4.05
4.04
2.97
2.94
2.90
2.89
2.86
2.61
2.60
2.59
2.58
2.13
1.16
1.03
0.34
0.17
0.06
TBDMSO
NH
O
N
S
OTBDMS
^{1}H NMR (360 MHz, CDCl$_3$)
0.97
1.00
1.04
1.10
1.21
1.04
1.04
3.55
2.09
2.98
6.07
9.13
9.29
6.35
3.15
3.03
f1 (ppm)

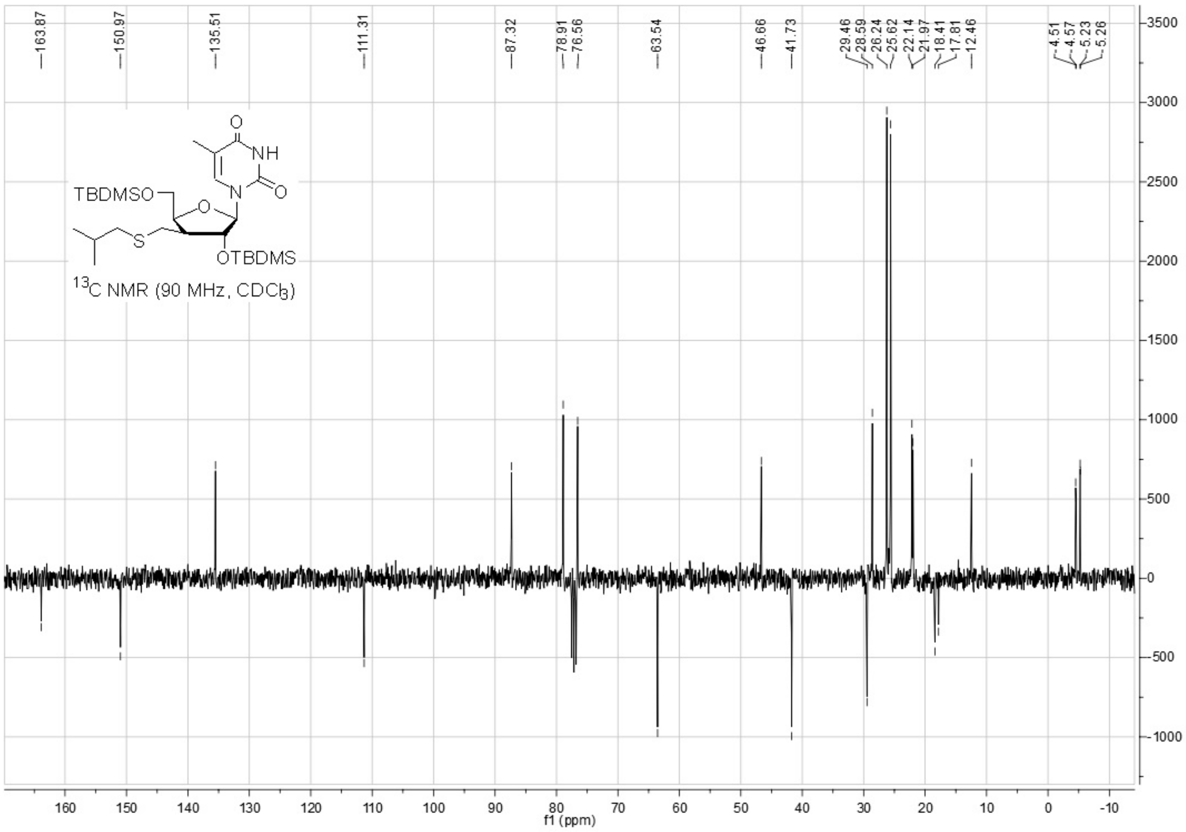

163.87
150.97
135.51
111.31
87.32
78.91
76.56
63.54
46.66
41.73
29.46
28.59
26.24
25.62
22.14
21.97
18.41
17.81
12.46
-4.51
-4.57
-5.23
-5.26
TBDMSO
NH
O
N
S
OTBDMS
^{13}C NMR (90 MHz, CDCl$_3$)
f1 (ppm)

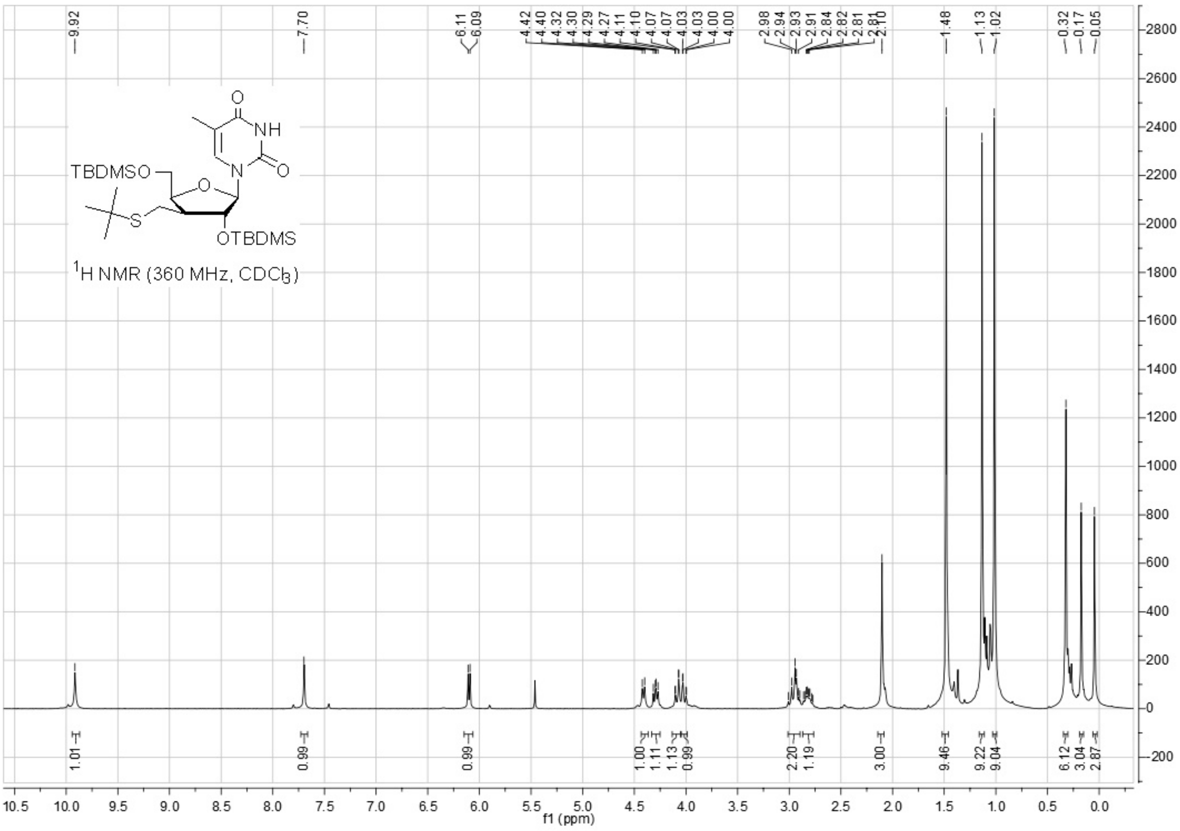

9.92
7.70
6.11
6.09
4.42
4.40
4.32
4.30
4.29
4.27
4.11
4.10
4.07
4.07
4.03
4.03
4.00
4.00
2.98
2.94
2.93
2.91
2.84
2.82
2.81
2.81
2.10
1.48
1.13
1.02
0.32
0.17
0.05
TBDMSO
O
NH
N
O
S
OTBDMS
^{1}H NMR (360 MHz, CDCl3)
1.01
0.99
0.99
1.00
1.11
1.13
0.99
2.20
1.19
3.00
9.46
9.22
9.04
6.12
3.04
2.87
f1 (ppm)

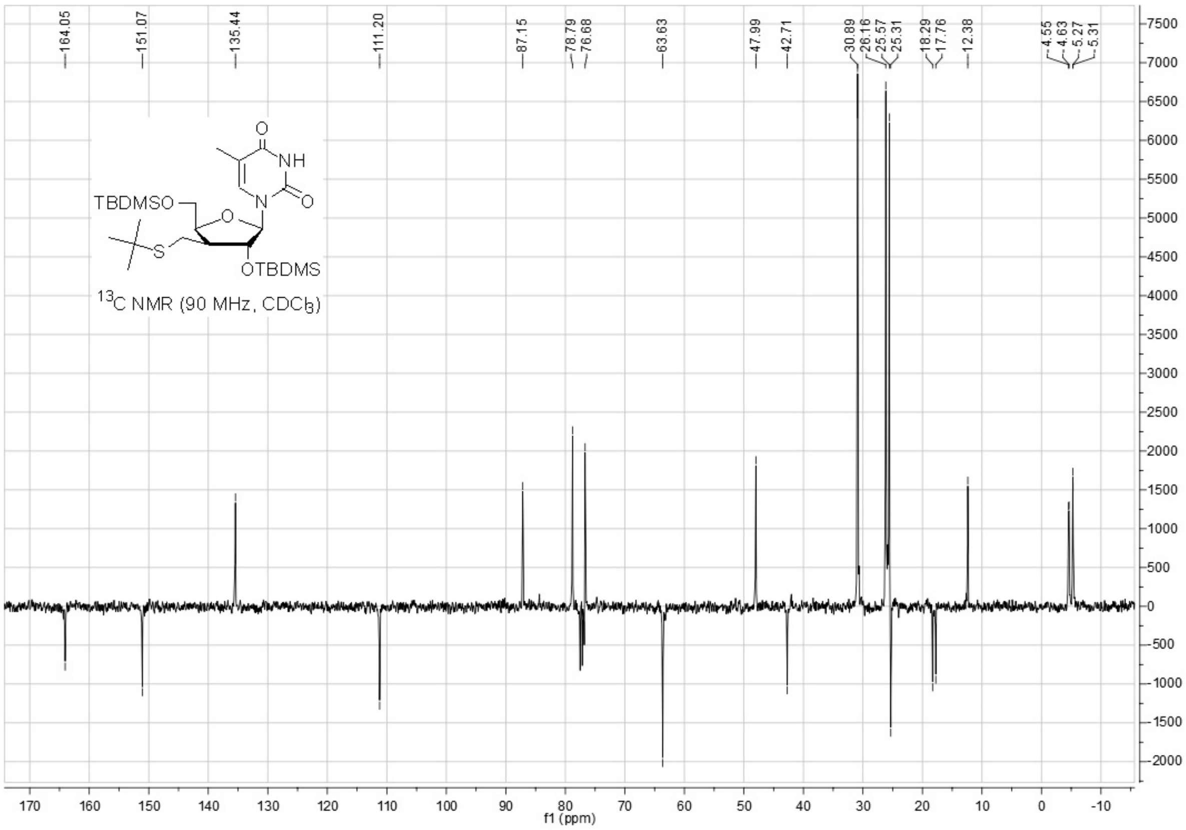

164.05
151.07
135.44
111.20
87.15
78.79
76.68
63.63
47.99
42.71
30.89
26.16
25.57
25.31
18.29
17.76
12.38
-4.55
-4.63
-5.27
-5.31
TBDMSO
O
NH
N
O
S
OTBDMS
^{13}C NMR (90 MHz, CDCl3)
f1 (ppm)

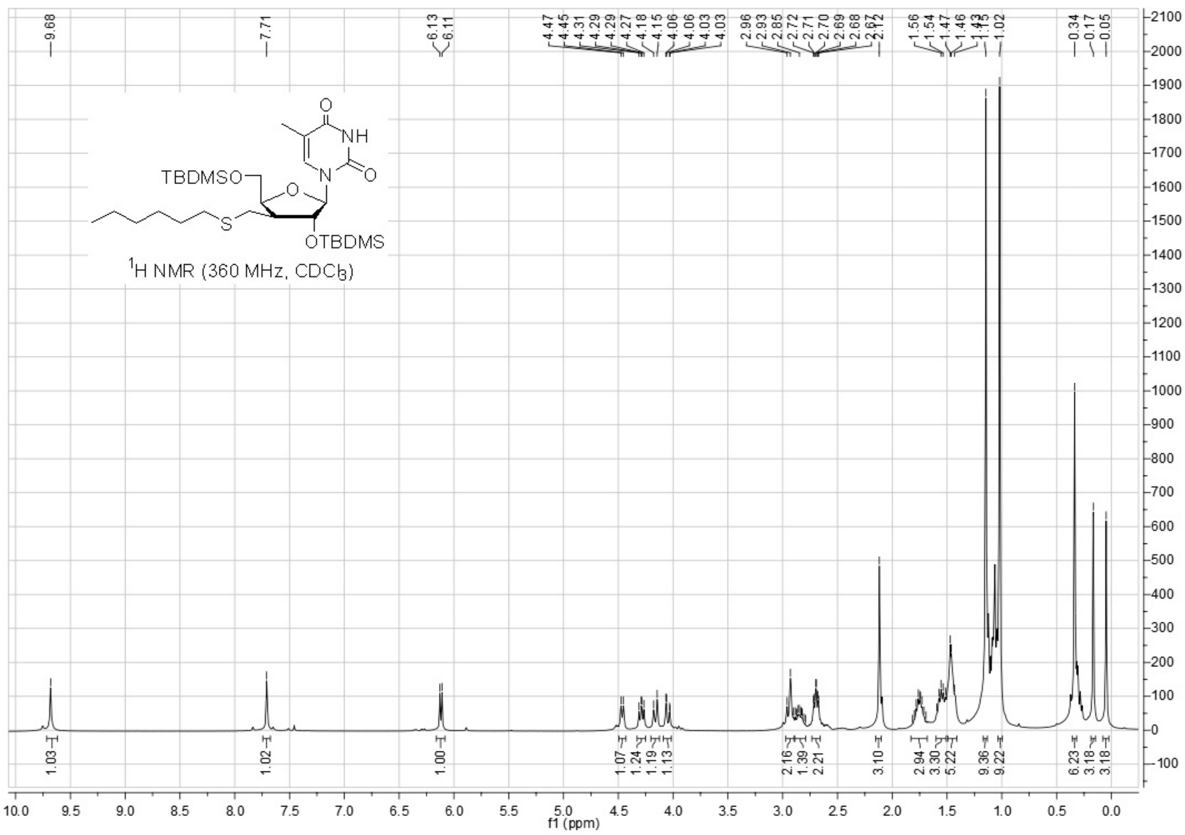
TBDMSO
NH
O
N
S
OTBDMS
^{1}H NMR (360 MHz, CDCl3)
f1 (ppm)

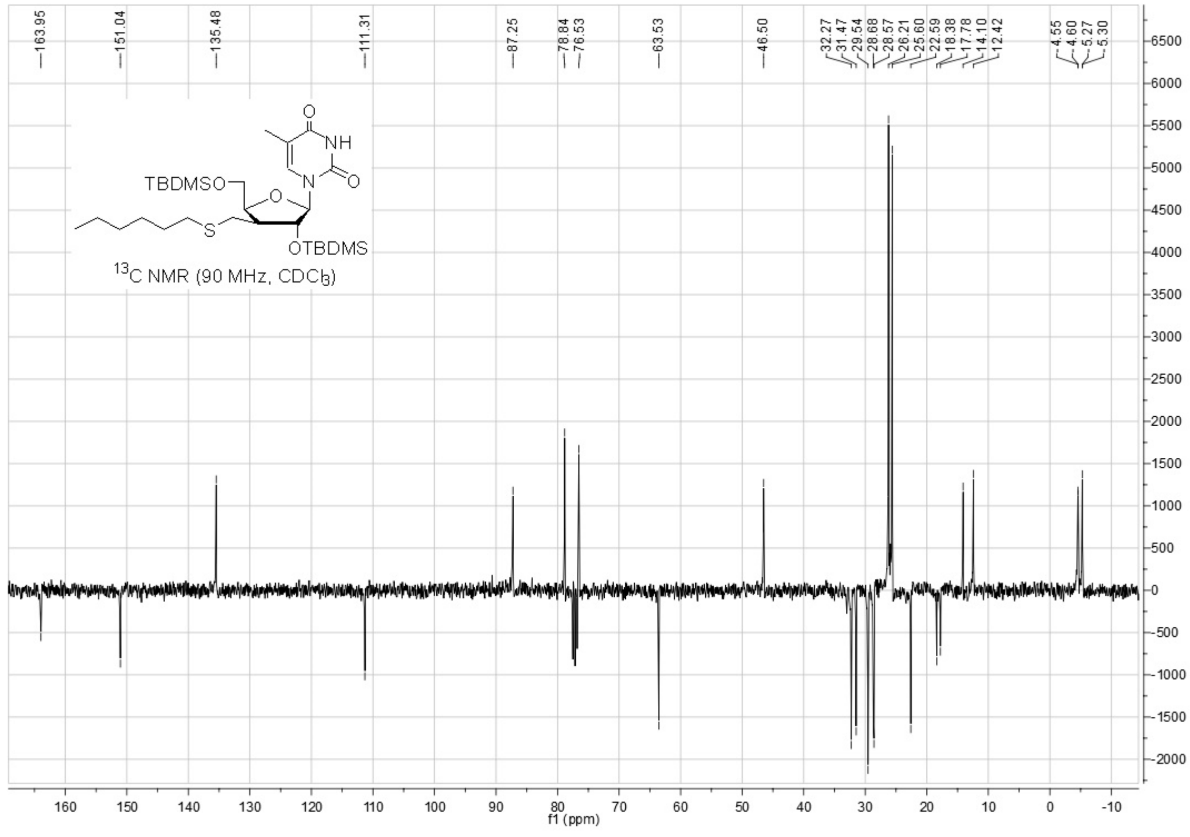
TBDMSO
NH
O
N
S
OTBDMS
^{13}C NMR (90 MHz, CDCl3)
f1 (ppm)

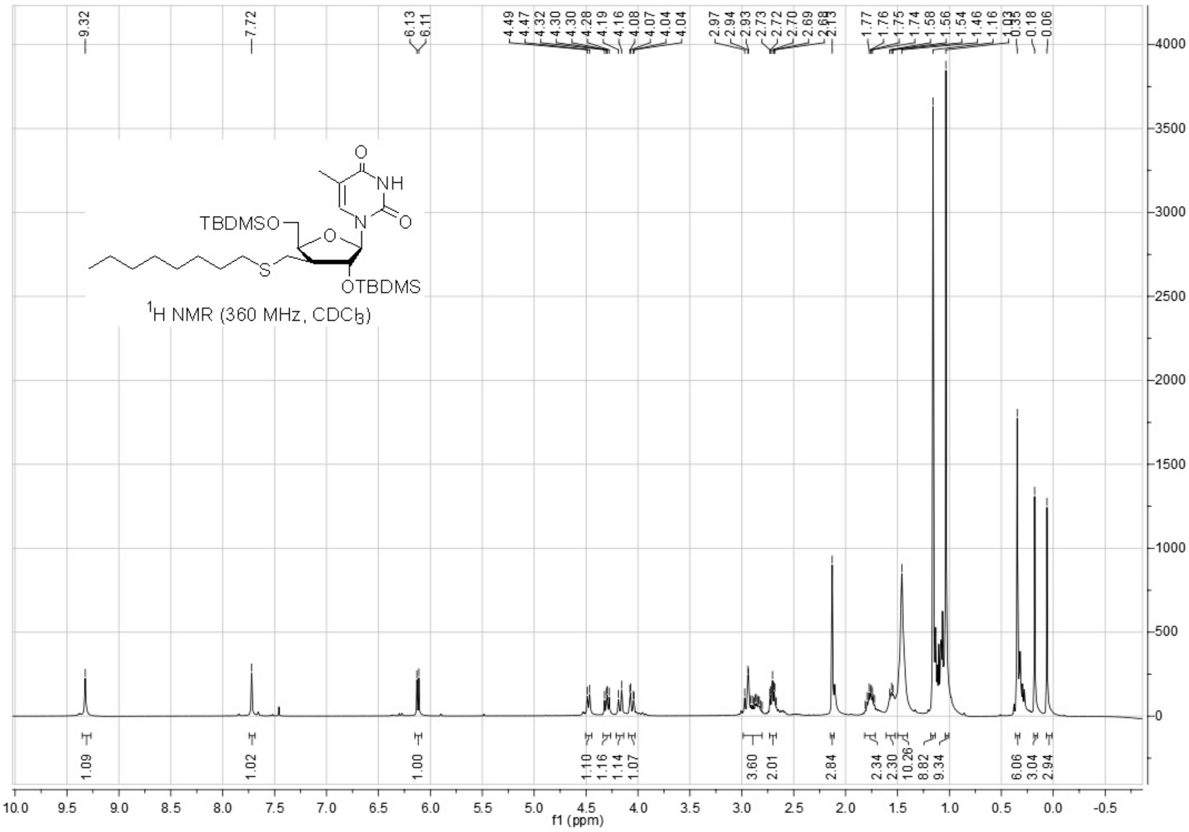

TBDMSO
O
NH
N
O
S
OTBDMS
^{1}H NMR (360 MHz, CDCl$_3$)
f1 (ppm)

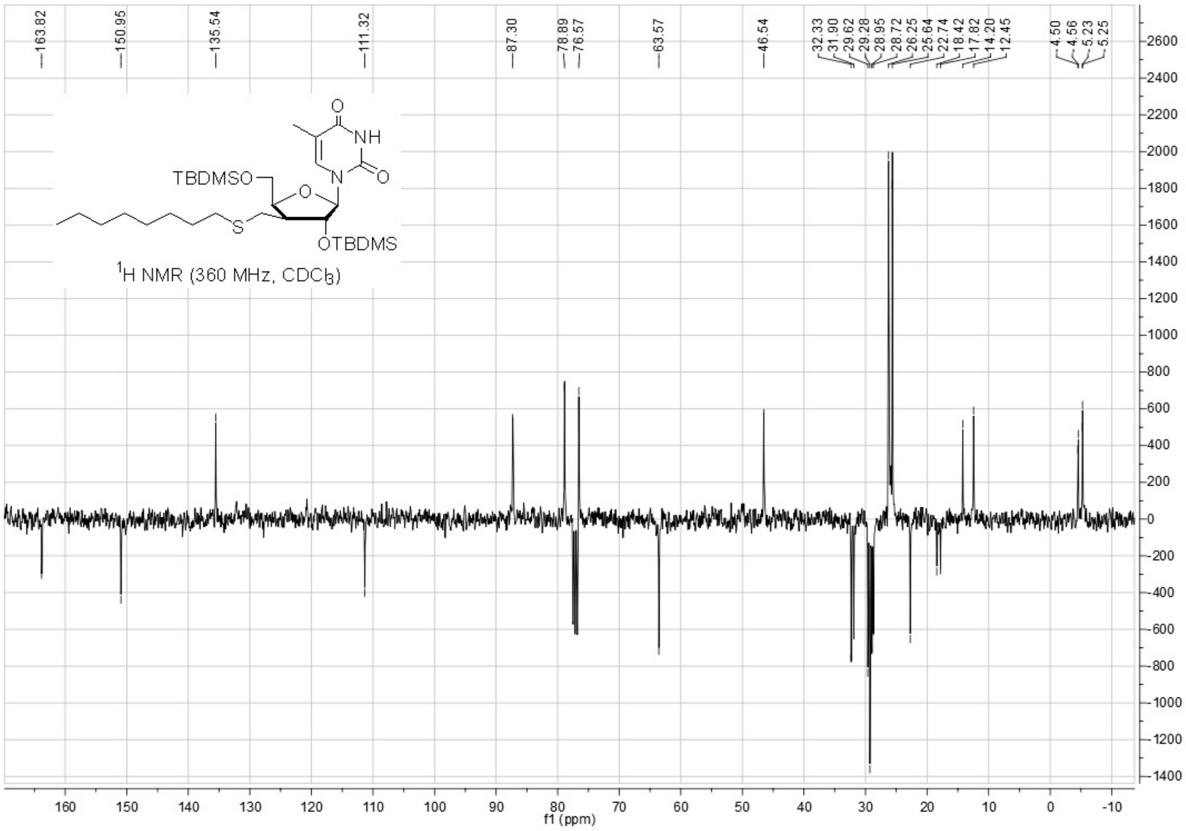

TBDMSO
O
NH
N
O
S
OTBDMS
^{1}H NMR (360 MHz, CDCl$_3$)
f1 (ppm)

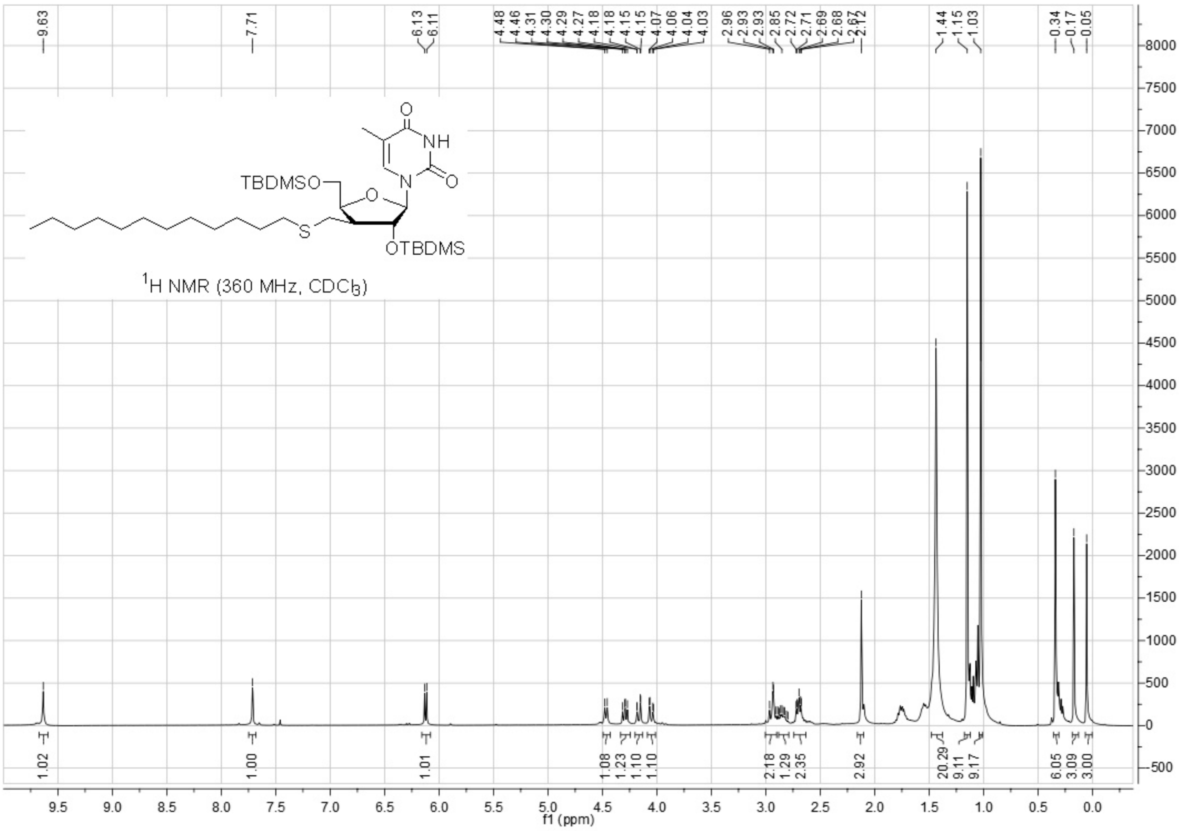
TBDMSO
NH
O
N
OTBDMS
S
1H NMR (360 MHz, CDCl3)
f1 (ppm)

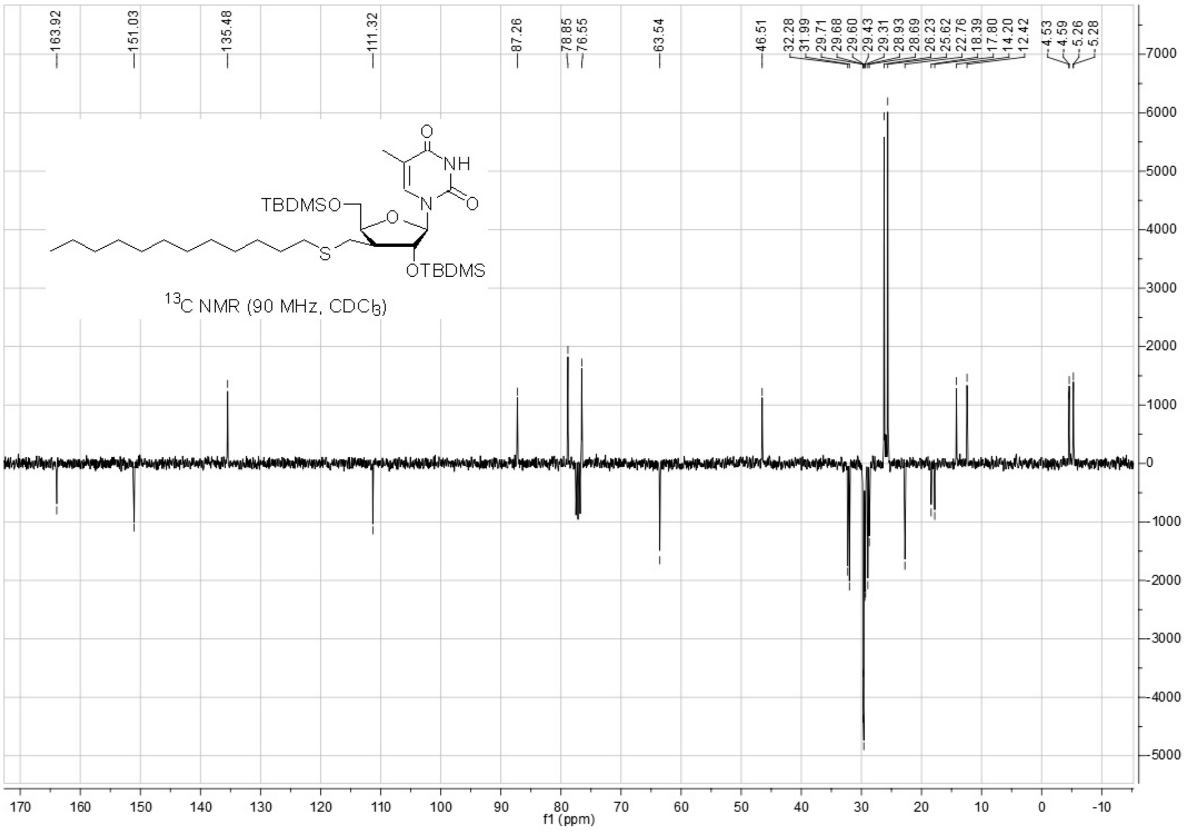
TBDMSO
NH
O
N
OTBDMS
S
13C NMR (90 MHz, CDCl3)
f1 (ppm)

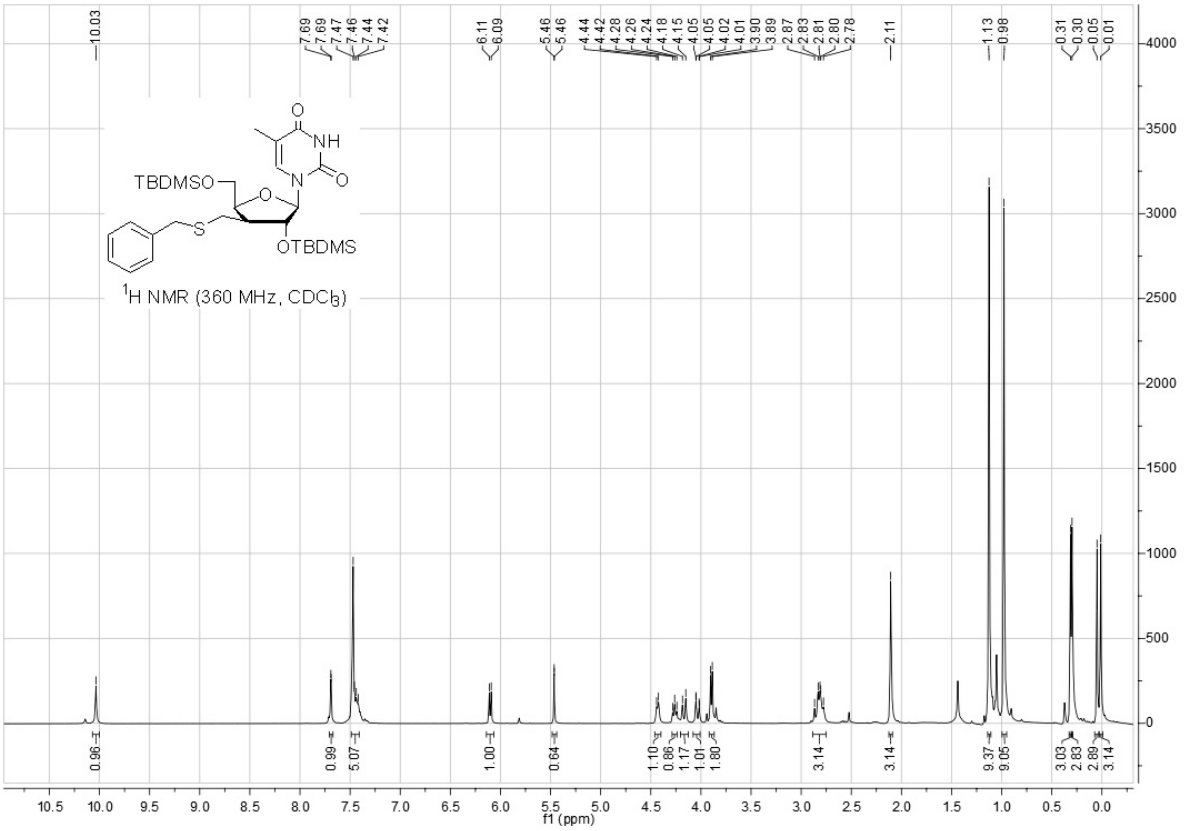

TBDMSO
S
O
NH
N
O
OTBDMS
1H NMR (360 MHz, CDCl3)
10.03
7.69
7.69
7.47
7.46
7.44
7.42
6.11
6.09
5.46
5.46
4.44
4.42
4.28
4.26
4.24
4.18
4.15
4.05
4.05
4.02
4.01
3.90
3.89
2.87
2.83
2.81
2.80
2.78
2.11
1.13
0.98
0.31
0.30
0.05
0.01
0.96
0.99
5.07
1.00
0.64
1.10
0.86
1.17
1.01
1.80
3.14
3.14
9.37
9.05
3.03
2.83
2.89
3.14
f1 (ppm)

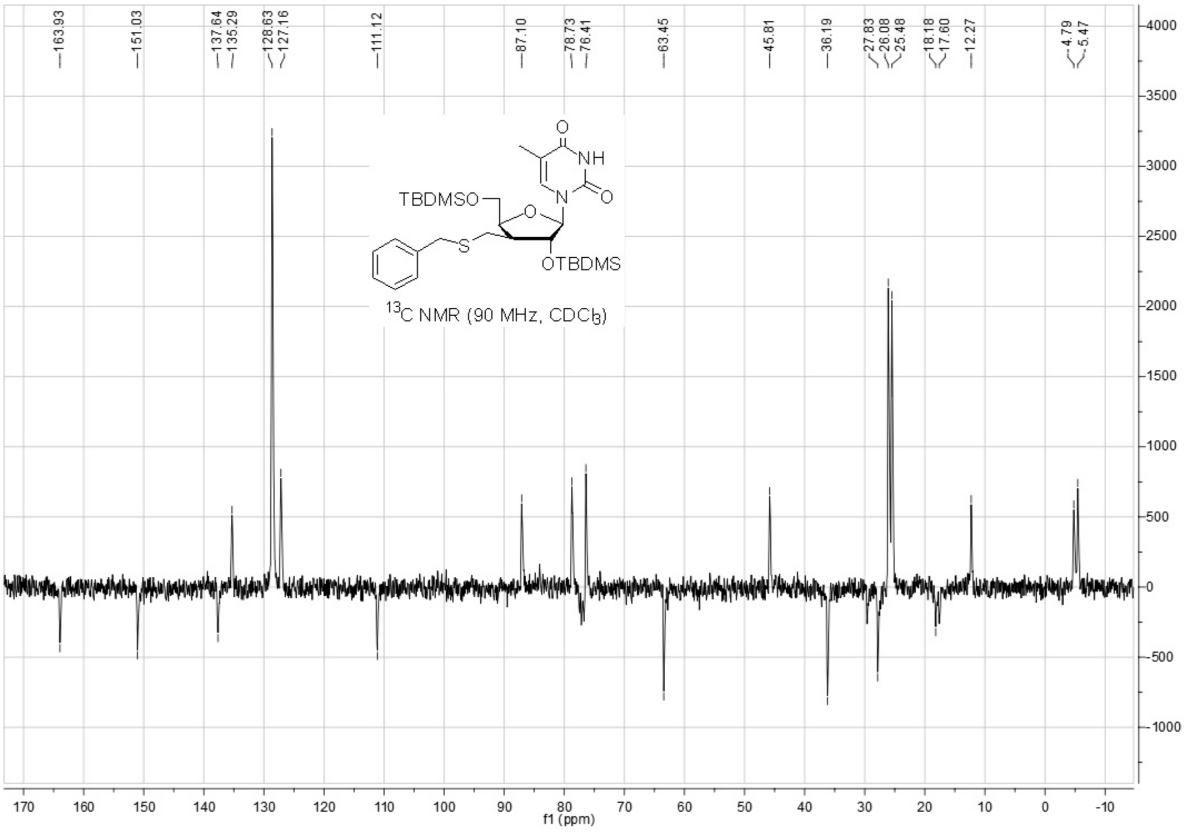

TBDMSO
S
O
NH
N
O
OTBDMS
13C NMR (90 MHz, CDCl3)
163.93
151.03
137.64
135.29
128.63
127.16
111.12
87.10
78.73
76.41
63.45
45.81
36.19
27.83
26.08
25.48
18.18
17.60
12.27
-4.79
-5.47
f1 (ppm)

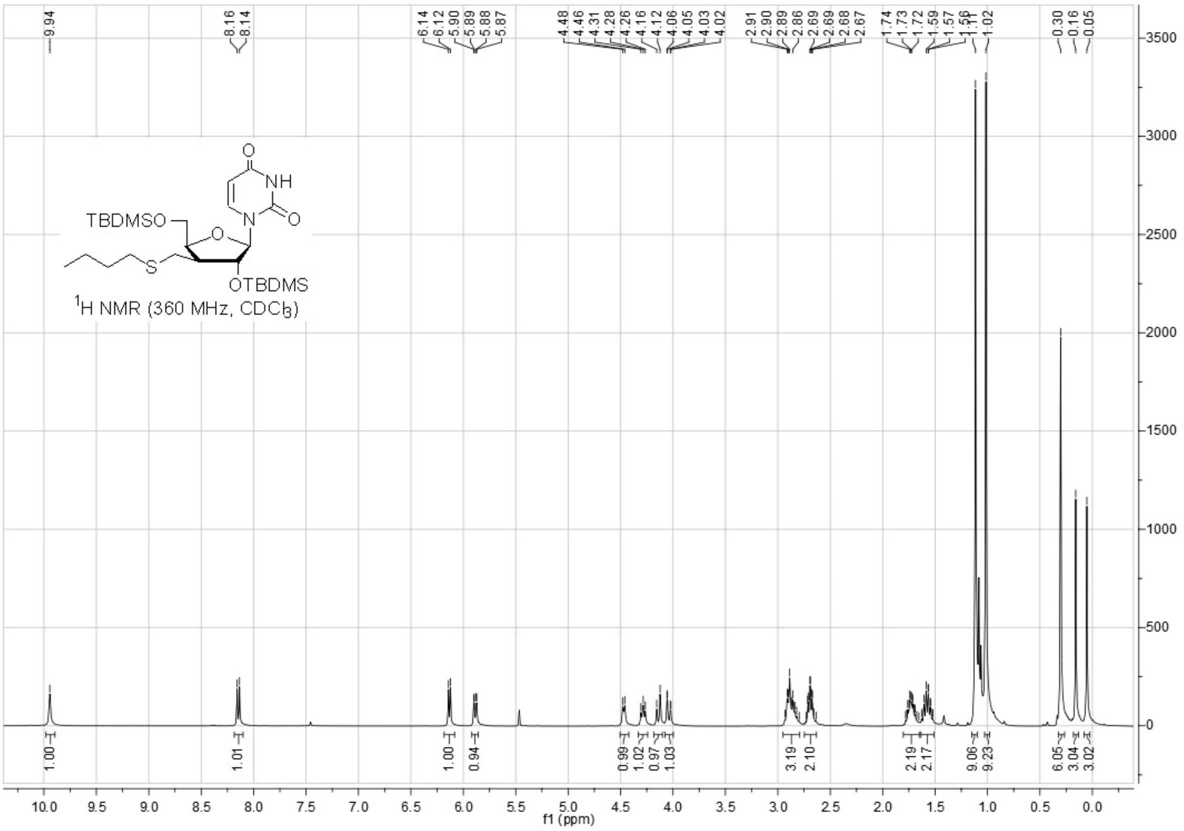

9.94
8.16
8.14
6.14
6.12
5.90
5.89
5.88
5.87
4.48
4.46
4.31
4.28
4.26
4.16
4.12
4.06
4.05
4.03
4.02
2.91
2.90
2.89
2.86
2.69
2.69
2.68
2.67
1.74
1.73
1.72
1.59
1.57
1.56
1.11
1.02
0.30
0.16
0.05
O
NH
TBDMSO
N
O
O
S
OTBDMS
^{1}H NMR (360 MHz, CDCl$_3$)
1.00
1.01
1.00
0.94
0.99
1.02
0.97
1.03
3.19
2.10
2.19
2.17
9.06
9.23
6.05
3.04
3.02
10.0 9.5 9.0 8.5 8.0 7.5 7.0 6.5 6.0 5.5 5.0 4.5 4.0 3.5 3.0 2.5 2.0 1.5 1.0 0.5 0.0
f1 (ppm)
3500 3000 2500 2000 1500 1000 500 0

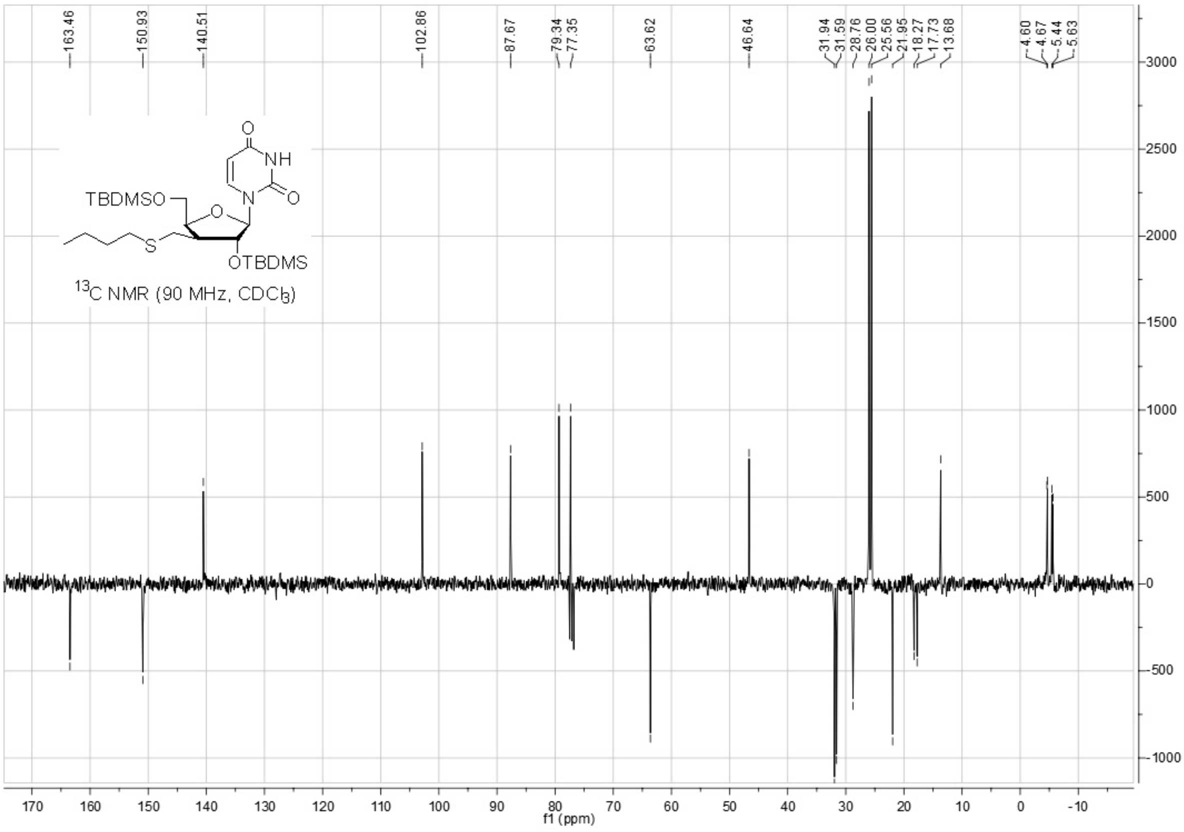

163.46
150.93
140.51
102.86
87.67
79.34
77.35
63.62
46.64
31.94
31.59
28.76
26.00
25.56
21.95
18.27
17.73
13.68
-4.60
-4.67
-5.44
-5.63
O
NH
TBDMSO
N
O
O
S
OTBDMS
^{13}C NMR (90 MHz, CDCl$_3$)
170 160 150 140 130 120 110 100 90 80 70 60 50 40 30 20 10 0 -10
f1 (ppm)
3000 2500 2000 1500 1000 500 0 -500 -1000

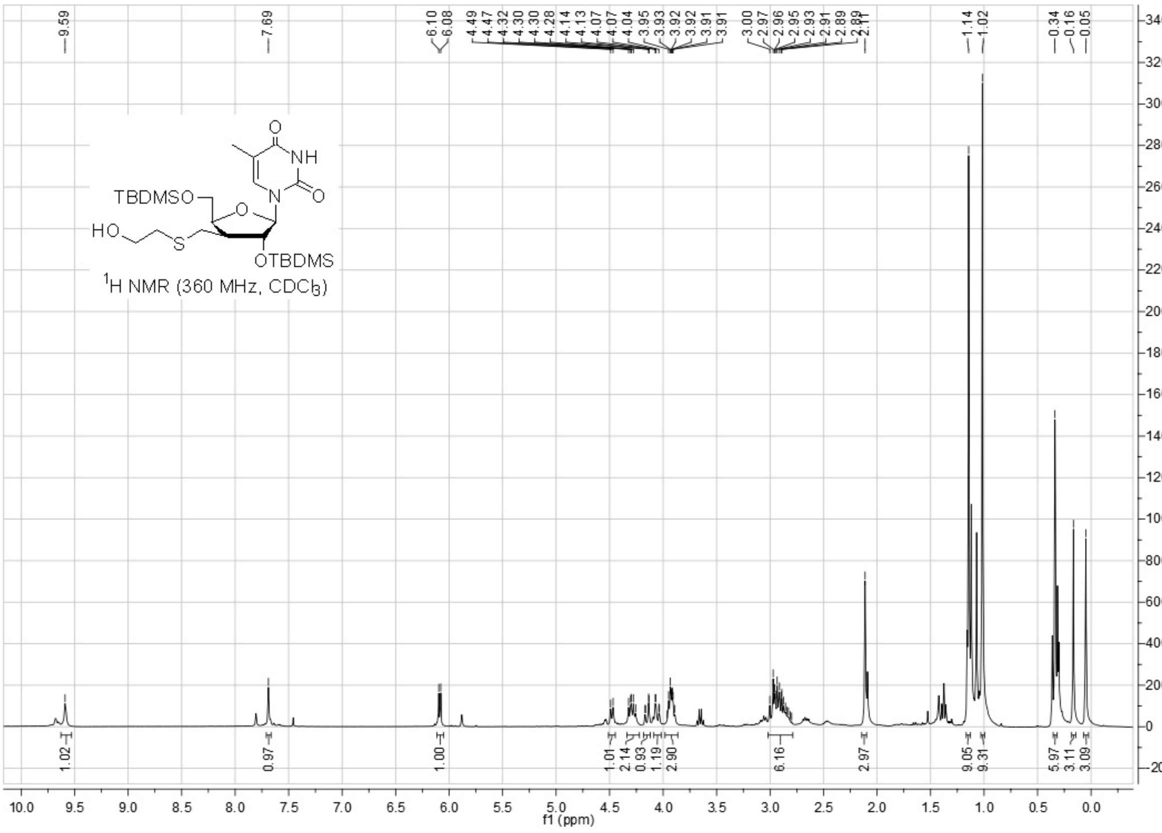
TBDMSO
HO
S
OTBDMS
NH
^{1}H NMR (360 MHz, CDCl$_3$)
f1 (ppm)

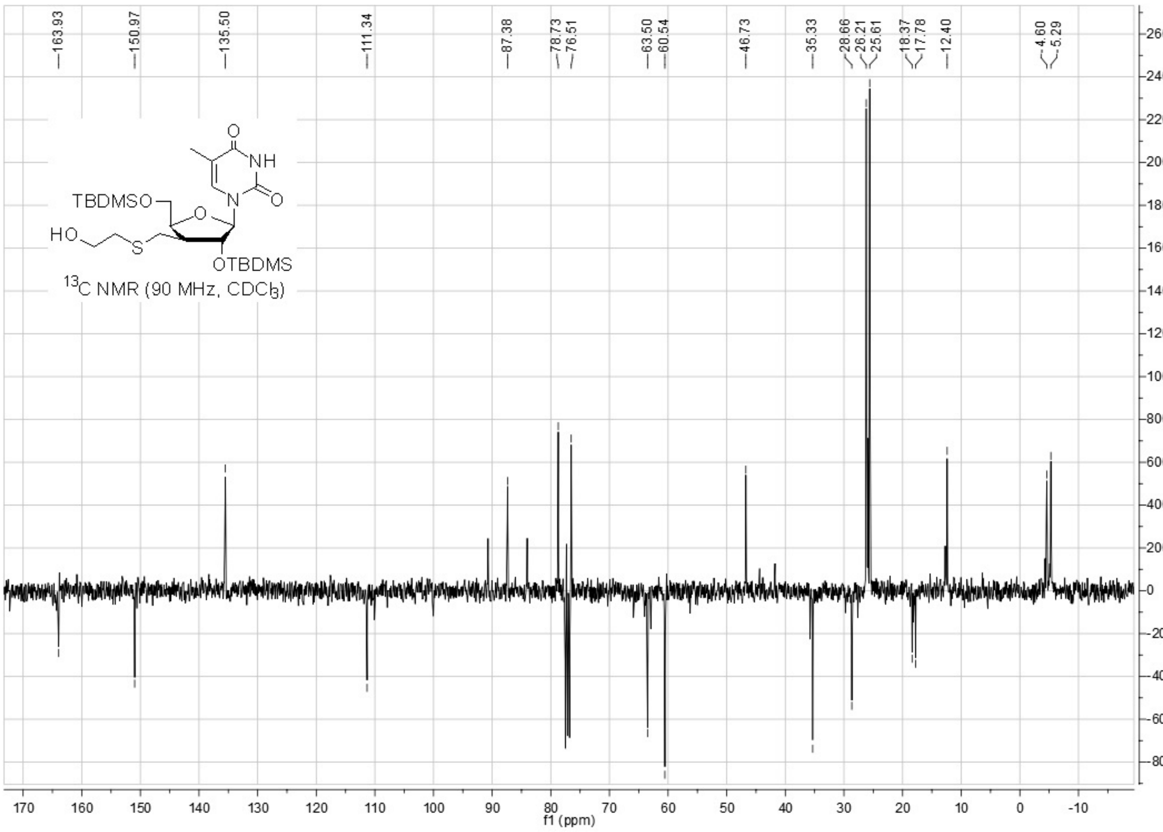
TBDMSO
HO
S
OTBDMS
NH
^{13}C NMR (90 MHz, CDCl$_3$)
f1 (ppm)

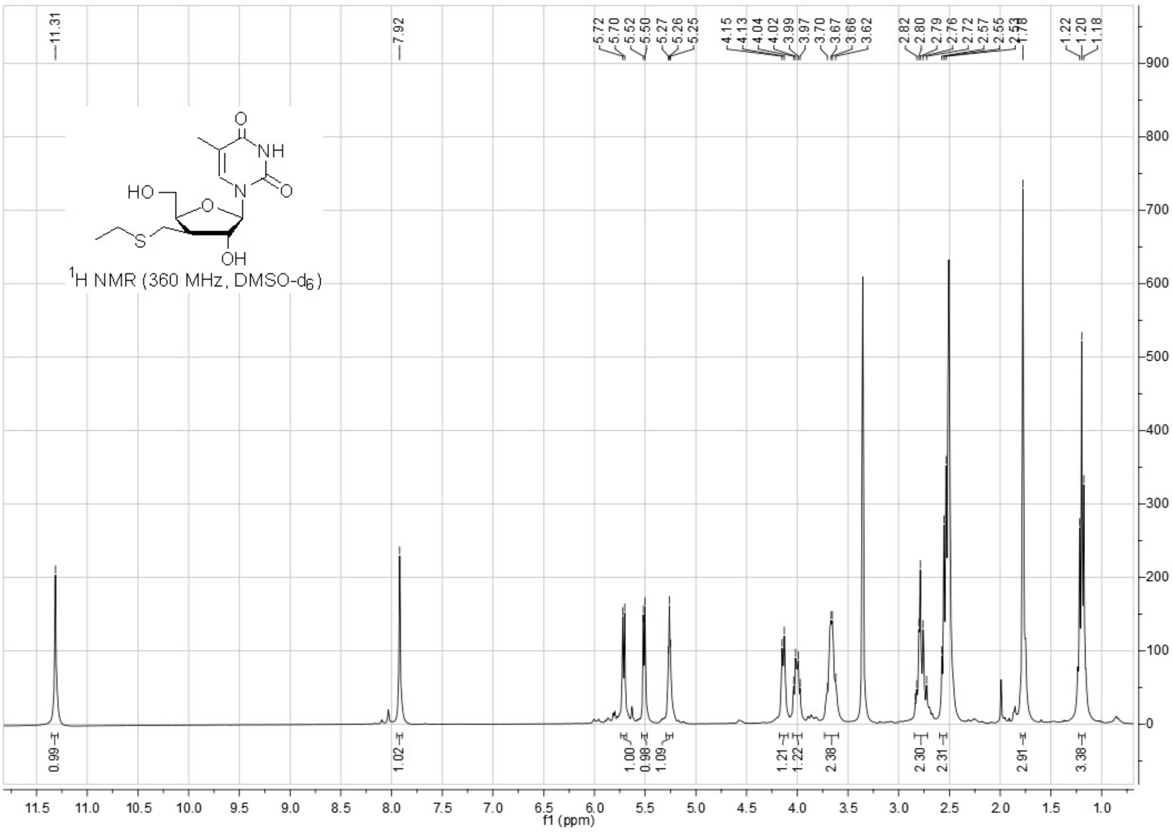

O
NH
N
O
HO
O
S
OH
1H NMR (360 MHz, DMSO-d6)
11.31
7.92
5.72
5.70
5.52
5.50
5.27
5.26
5.25
4.15
4.13
4.04
4.02
3.99
3.97
3.70
3.67
3.66
3.62
2.82
2.80
2.79
2.76
2.72
2.57
2.55
1.78
1.22
1.20
1.18
0.99
1.02
1.00
0.98
1.09
1.21
1.22
2.38
2.30
2.31
2.91
3.38
f1 (ppm)

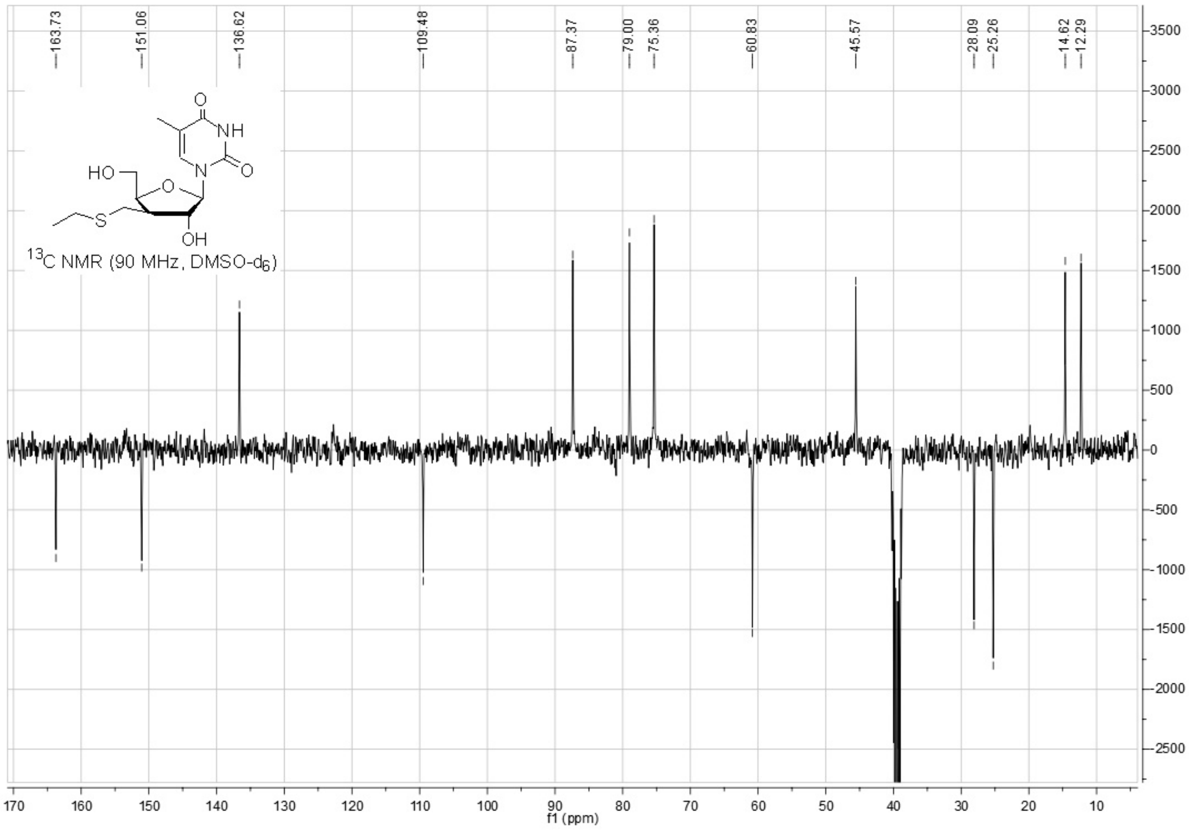

O
NH
N
O
HO
O
S
OH
13C NMR (90 MHz, DMSO-d6)
163.73
151.06
136.62
109.48
87.37
79.00
75.36
60.83
45.57
28.09
25.26
14.62
12.29
f1 (ppm)
